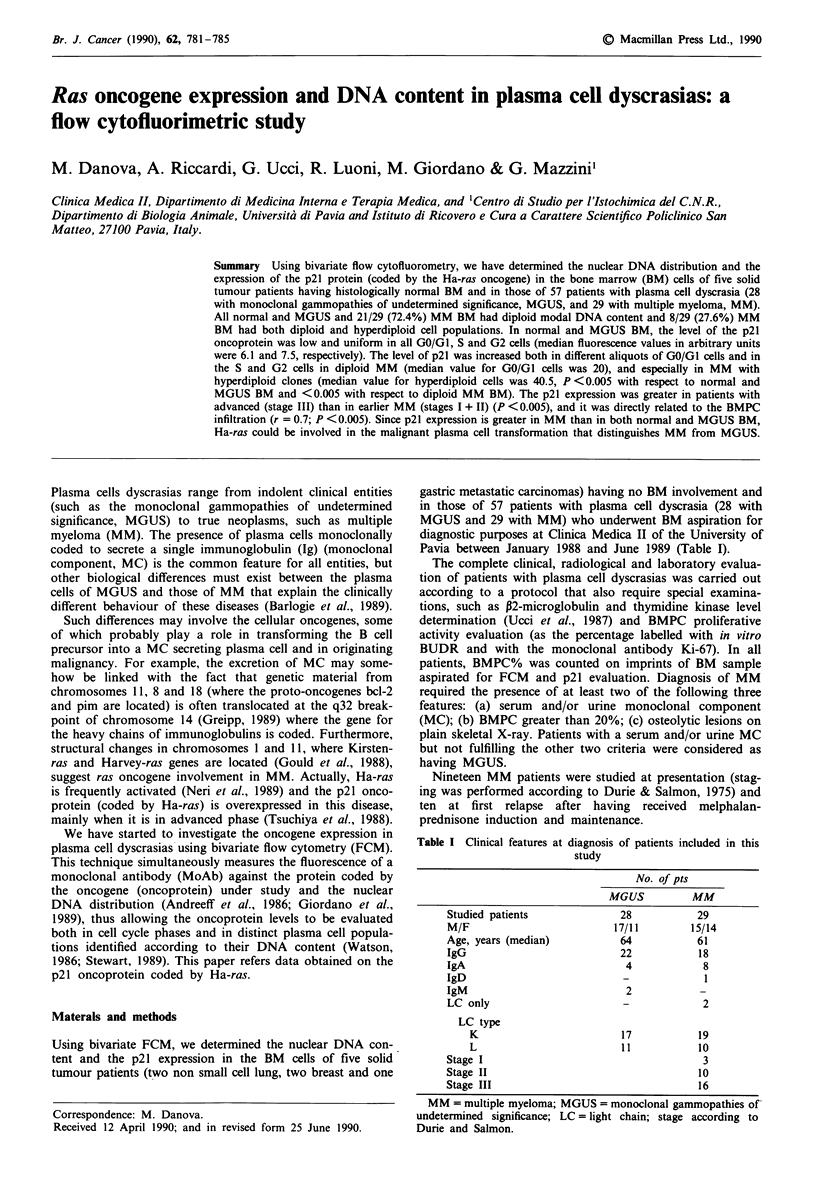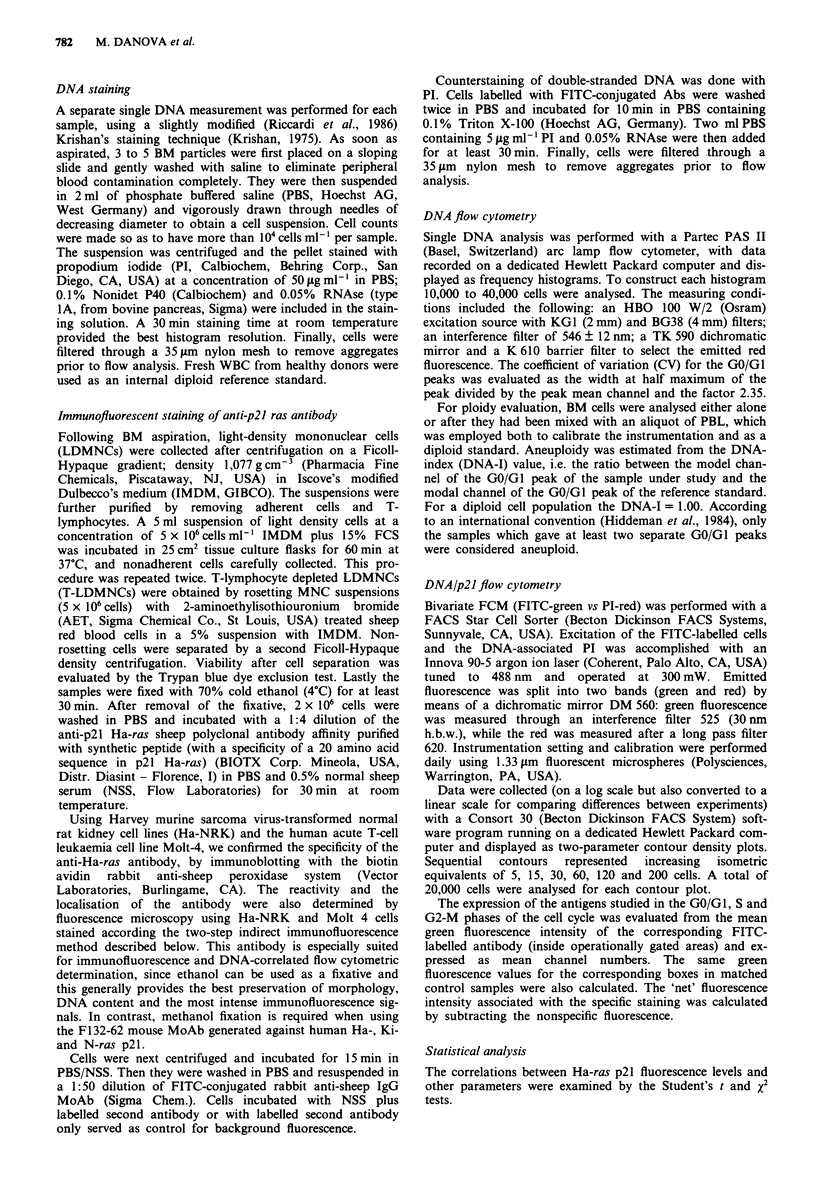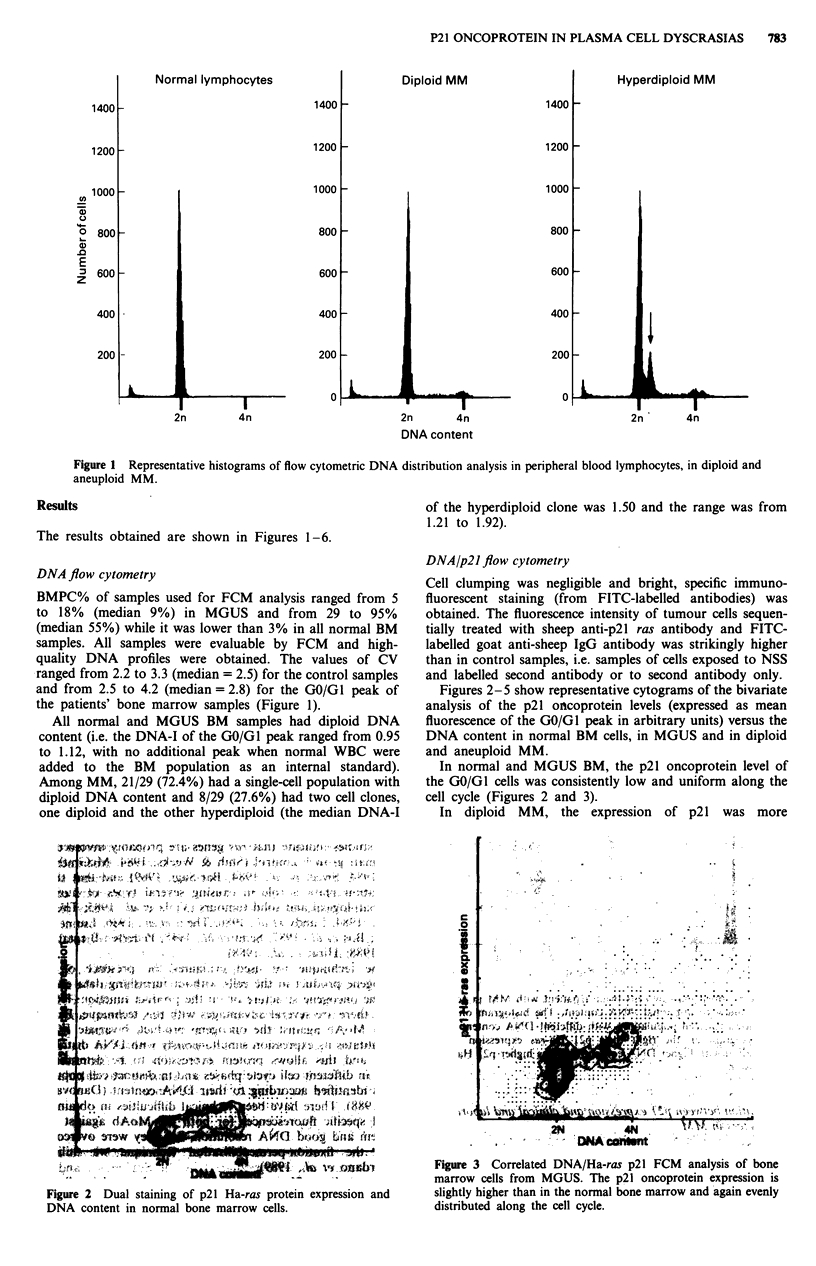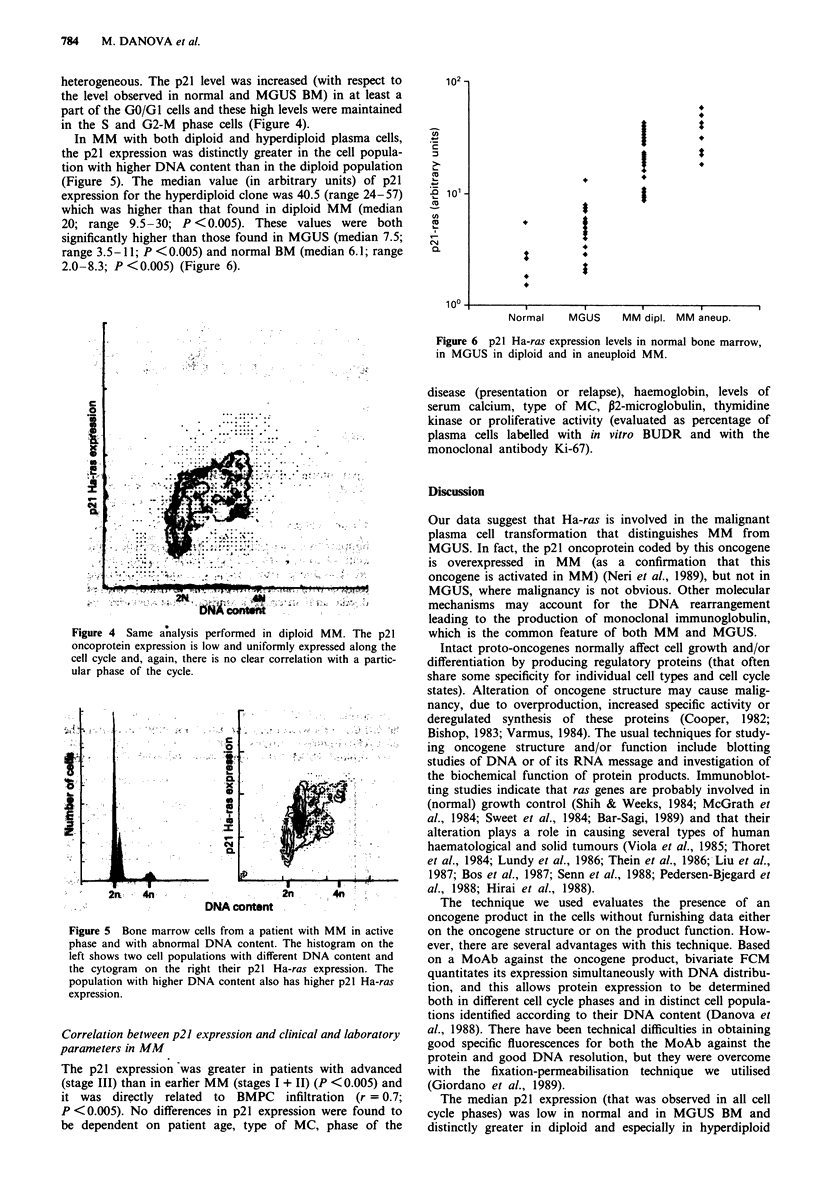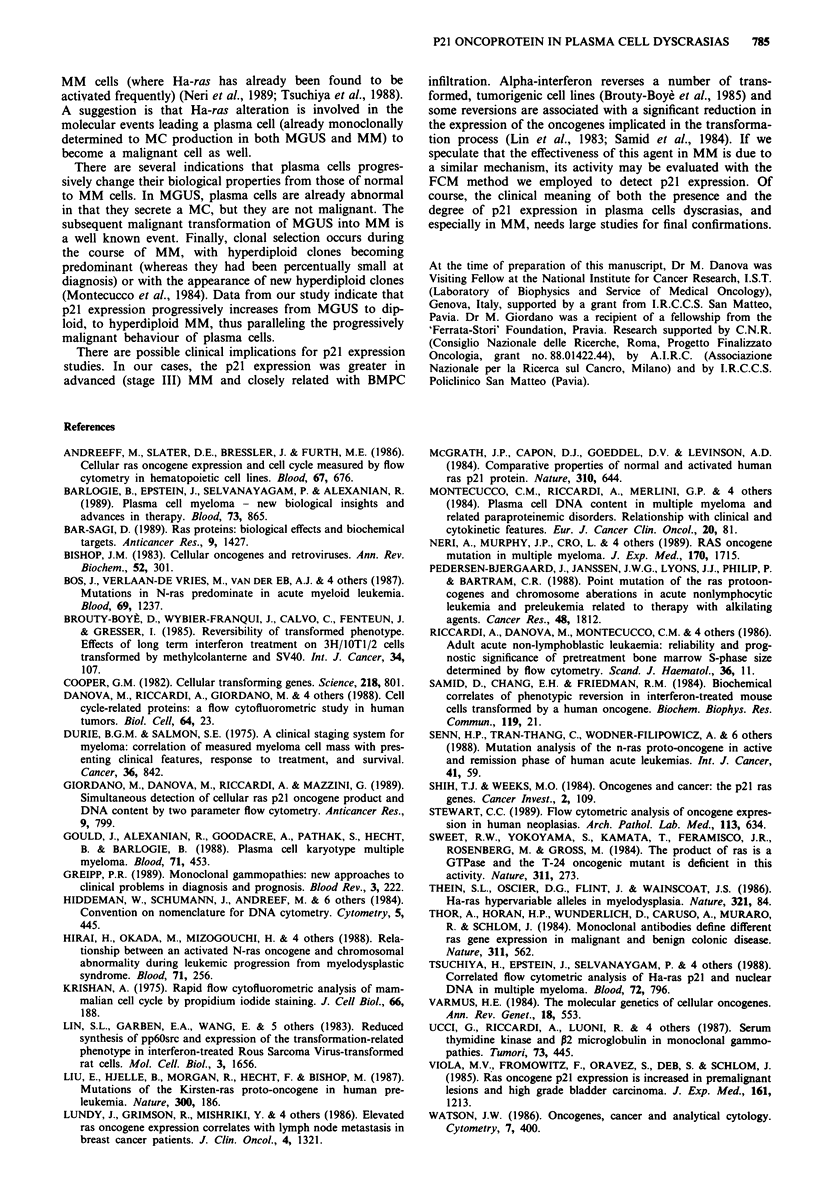# Ras oncogene expression and DNA content in plasma cell dyscrasias: a flow cytofluorimetric study.

**DOI:** 10.1038/bjc.1990.379

**Published:** 1990-11

**Authors:** M. Danova, A. Riccardi, G. Ucci, R. Luoni, M. Giordano, G. Mazzini

**Affiliations:** Dipartimento di Medicina Interna e Terapia Medica, Università di Pavia, Italy.

## Abstract

Using bivariate flow cytofluorometry, we have determined the nuclear DNA distribution and the expression of the p21 protein (coded by the Ha-ras oncogene) in the bone marrow (BM) cells of five solid tumour patients having histologically normal BM and in those of 57 patients with plasma cell dyscrasia (28 with monoclonal gammopathies of undertermined significance, MGUS, and 29 with multiple myeloma, MM). All normal and MGUS and 21/29 (72.4%) MM BM had diploid modal DNA content and 8/29 (27.6%) MM BM had both diploid and hyperdiploid cell populations. In normal and MGUS BM, the level of the p21 oncoprotein was low and uniform in all G0/G1, S and G2 cells (median fluorescence values in arbitrary units were 6.1 and 7.5, respectively). The level of p21 was increased both in different aliquots of G0/G1 cells and in the S and G2 cells in diploid MM (median value for G0/G1 cells was 20), and especially in MM with hyperdiploid clones (median value for hyperdiploid cells was 40.5, P less than 0.005 with respect to normal and MGUS BM and less than 0.005 with respect to diploid MM BM). The p21 expression was greater in patients with advanced (stage III) than in earlier MM (stages I + II) (P less than 0.005), and it was directly related to the BMPC infiltration (r = 0.7; P less than 0.005). Since p21 expression is greater in MM than in both normal and MGUS BM, Ha-ras could be involved in the malignant plasma cell transformation that distinguishes MM from MGUS.


Br. J. Cancer (1990), 62, 781-785 © Macmillan Press Ltd., 1990

### *Ras* oncogene expression and DNA content in plasma cell dyscrasias: a flow cytofluorimetric study

M. Danova, A. Riccardi, G. Ucci, R. Luoni, M. Giordano & G. Mazzini[1]

*Clinica Medica II, Dipartimento di Medicina Interna e Terapia Medica, and [1]Centro di Studio per l'Istochimica del C.N.R., Dipartimento di Biologia Animale, Università di Pavia and Istituto di Ricovero e Cura a Carattere Scientifico Policlinico San Matteo, 27100 Pavia, Italy.*

**Summary** Using bivariate flow cytofluorometry, we have determined the nuclear DNA distribution and the expression of the p21 protein (coded by the Ha-*ras* oncogene) in the bone marrow (BM) cells of five solid tumour patients having histologically normal BM and in those of 57 patients with plasma cell dyscrasia (28 with monoclonal gammopathies of undetermined significance, MGUS, and 29 with multiple myeloma, MM). All normal and MGUS and 21/29 (72.4%) MM BM had diploid modal DNA content and 8/29 (27.6%) MM BM had both diploid and hyperdiploid cell populations. In normal and MGUS BM, the level of the p21 oncoprotein was low and uniform in all G0/G1, S and G2 cells (median fluorescence values in arbitrary units were 6.1 and 7.5, respectively). The level of p21 was increased both in different aliquots of G0/G1 cells and in the S and G2 cells in diploid MM (median value for G0/G1 cells was 20), and especially in MM with hyperdiploid clones (median value for hyperdiploid cells was 40.5, $P < 0.005$ with respect to normal and MGUS BM and $< 0.005$ with respect to diploid MM BM). The p21 expression was greater in patients with advanced (stage III) than in earlier MM (stages I + II) ($P < 0.005$), and it was directly related to the BMPC infiltration ($r = 0.7$; $P < 0.005$). Since p21 expression is greater in MM than in both normal and MGUS BM, Ha-*ras* could be involved in the malignant plasma cell transformation that distinguishes MM from MGUS.

Plasma cells dyscrasias range from indolent clinical entities (such as the monoclonal gammopathies of undetermined significance, MGUS) to true neoplasms, such as multiple myeloma (MM). The presence of plasma cells monoclonally coded to secrete a single immunoglobulin (Ig) (monoclonal component, MC) is the common feature for all entities, but other biological differences must exist between the plasma cells of MGUS and those of MM that explain the clinically different behaviour of these diseases (Barlogie *et al.*, 1989).

Such differences may involve the cellular oncogenes, some of which probably play a role in transforming the B cell precursor into a MC secreting plasma cell and in originating malignancy. For example, the excretion of MC may somehow be linked with the fact that genetic material from chromosomes 11, 8 and 18 (where the proto-oncogenes bcl-2 and pim are located) is often translocated at the q32 breakpoint of chromosome 14 (Greipp, 1989) where the gene for the heavy chains of immunoglobulins is coded. Furthermore, structural changes in chromosomes 1 and 11, where Kirsten-*ras* and Harvey-*ras* genes are located (Gould *et al.*, 1988), suggest *ras* oncogene involvement in MM. Actually, Ha-*ras* is frequently activated (Neri *et al.*, 1989) and the p21 oncoprotein (coded by Ha-*ras*) is overexpressed in this disease, mainly when it is in advanced phase (Tsuchiya *et al.*, 1988).

We have started to investigate the oncogene expression in plasma cell dyscrasias using bivariate flow cytometry (FCM). This technique simultaneously measures the fluorescence of a monoclonal antibody (MoAb) against the protein coded by the oncogene (oncoprotein) under study and the nuclear DNA distribution (Andreeff *et al.*, 1986; Giordano *et al.*, 1989), thus allowing the oncoprotein levels to be evaluated both in cell cycle phases and in distinct plasma cell populations identified according to their DNA content (Watson, 1986; Stewart, 1989). This paper refers data obtained on the p21 oncoprotein coded by Ha-*ras*.

### Materals and methods

Using bivariate FCM, we determined the nuclear DNA content and the p21 expression in the BM cells of five solid tumour patients (two non small cell lung, two breast and one gastric metastatic carcinomas) having no BM involvement and in those of 57 patients with plasma cell dyscrasia (28 with MGUS and 29 with MM) who underwent BM aspiration for diagnostic purposes at Clinica Medica II of the University of Pavia between January 1988 and June 1989 (Table I).

The complete clinical, radiological and laboratory evaluation of patients with plasma cell dyscrasias was carried out according to a protocol that also require special examinations, such as β2-microglobulin and thymidine kinase level determination (Ucci *et al.*, 1987) and BMPC proliferative activity evaluation (as the percentage labelled with *in vitro* BUDR and with the monoclonal antibody Ki-67). In all patients, BMPC% was counted on imprints of BM sample aspirated for FCM and p21 evaluation. Diagnosis of MM required the presence of at least two of the following three features: (a) serum and/or urine monoclonal component (MC); (b) BMPC greater than 20%; (c) osteolytic lesions on plain skeletal X-ray. Patients with a serum and/or urine MC but not fulfilling the other two criteria were considered as having MGUS.

Nineteen MM patients were studied at presentation (staging was performed according to Durie & Salmon, 1975) and ten at first relapse after having received melphalan-prednisone induction and maintenance.

**Table I** Clinical features at diagnosis of patients included in this study

| | *No. of pts* | |
|---|---|---|
| | *MGUS* | *MM* |
| Studied patients | 28 | 29 |
| M/F | 17/11 | 15/14 |
| Age, years (median) | 64 | 61 |
| IgG | 22 | 18 |
| IgA | 4 | 8 |
| IgD | – | 1 |
| IgM | 2 | – |
| LC only | – | 2 |
| LC type | | |
| K | 17 | 19 |
| L | 11 | 10 |
| Stage I | | 3 |
| Stage II | | 10 |
| Stage III | | 16 |

MM = multiple myeloma; MGUS = monoclonal gammopathies of undetermined significance; LC = light chain; stage according to Durie and Salmon.

Correspondence: M. Danova.
Received 12 April 1990; and in revised form 25 June 1990.

*DNA staining*

A separate single DNA measurement was performed for each sample, using a slightly modified (Riccardi *et al*., 1986) Krishan's staining technique (Krishan, 1975). As soon as aspirated, 3 to 5 BM particles were first placed on a sloping slide and gently washed with saline to eliminate peripheral blood contamination completely. They were then suspended in 2 ml of phosphate buffered saline (PBS, Hoechst AG, West Germany) and vigorously drawn through needles of decreasing diameter to obtain a cell suspension. Cell counts were made so as to have more than $10^4$ cells $ml^{-1}$ per sample. The suspension was centrifuged and the pellet stained with propodium iodide (PI, Calbiochem, Behring Corp., San Diego, CA, USA) at a concentration of 50 $\mu g\ ml^{-1}$ in PBS; 0.1% Nonidet P40 (Calbiochem) and 0.05% RNAse (type 1A, from bovine pancreas, Sigma) were included in the staining solution. A 30 min staining time at room temperature provided the best histogram resolution. Finally, cells were filtered through a 35 $\mu$m nylon mesh to remove aggregates prior to flow analysis. Fresh WBC from healthy donors were used as an internal diploid reference standard.

*Immunofluorescent staining of anti-p21 ras antibody*

Following BM aspiration, light-density mononuclear cells (LDMNCs) were collected after centrifugation on a Ficoll-Hypaque gradient; density 1,077 g $cm^{-3}$ (Pharmacia Fine Chemicals, Piscataway, NJ, USA) in Iscove's modified Dulbecco's medium (IMDM, GIBCO). The suspensions were further purified by removing adherent cells and T-lymphocytes. A 5 ml suspension of light density cells at a concentration of $5 \times 10^6$ cells $ml^{-1}$ IMDM plus 15% FCS was incubated in 25 $cm^2$ tissue culture flasks for 60 min at 37°C, and nonadherent cells carefully collected. This procedure was repeated twice. T-lymphocyte depleted LDMNCs (T-LDMNCs) were obtained by rosetting MNC suspensions ($5 \times 10^6$ cells) with 2-aminoethylisothiouronium bromide (AET, Sigma Chemical Co., St Louis, USA) treated sheep red blood cells in a 5% suspension with IMDM. Non-rosetting cells were separated by a second Ficoll-Hypaque density centrifugation. Viability after cell separation was evaluated by the Trypan blue dye exclusion test. Lastly the samples were fixed with 70% cold ethanol (4°C) for at least 30 min. After removal of the fixative, $2 \times 10^6$ cells were washed in PBS and incubated with a 1:4 dilution of the anti-p21 Ha-*ras* sheep polyclonal antibody affinity purified with synthetic peptide (with a specificity of a 20 amino acid sequence in p21 Ha-*ras*) (BIOTX Corp. Mineola, USA, Distr. Diasint – Florence, I) in PBS and 0.5% normal sheep serum (NSS, Flow Laboratories) for 30 min at room temperature.

Using Harvey murine sarcoma virus-transformed normal rat kidney cell lines (Ha-NRK) and the human acute T-cell leukaemia cell line Molt-4, we confirmed the specificity of the anti-Ha-*ras* antibody, by immunoblotting with the biotin avidin rabbit anti-sheep peroxidase system (Vector Laboratories, Burlingame, CA). The reactivity and the localisation of the antibody were also determined by fluorescence microscopy using Ha-NRK and Molt 4 cells stained according the two-step indirect immunofluorescence method described below. This antibody is especially suited for immunofluorescence and DNA-correlated flow cytometric determination, since ethanol can be used as a fixative and this generally provides the best preservation of morphology, DNA content and the most intense immunofluorescence signals. In contrast, methanol fixation is required when using the F132-62 mouse MoAb generated against human Ha-, Ki- and N-*ras* p21.

Cells were next centrifuged and incubated for 15 min in PBS/NSS. Then they were washed in PBS and resuspended in a 1:50 dilution of FITC-conjugated rabbit anti-sheep IgG MoAb (Sigma Chem.). Cells incubated with NSS plus labelled second antibody or with labelled second antibody only served as control for background fluorescence.

Counterstaining of double-stranded DNA was done with PI. Cells labelled with FITC-conjugated Abs were washed twice in PBS and incubated for 10 min in PBS containing 0.1% Triton X-100 (Hoechst AG, Germany). Two ml PBS containing 5 $\mu g\ ml^{-1}$ PI and 0.05% RNAse were then added for at least 30 min. Finally, cells were filtered through a 35 $\mu$m nylon mesh to remove aggregates prior to flow analysis.

*DNA flow cytometry*

Single DNA analysis was performed with a Partec PAS II (Basel, Switzerland) arc lamp flow cytometer, with data recorded on a dedicated Hewlett Packard computer and displayed as frequency histograms. To construct each histogram 10,000 to 40,000 cells were analysed. The measuring conditions included the following: an HBO 100 W/2 (Osram) excitation source with KG1 (2 mm) and BG38 (4 mm) filters; an interference filter of 546 ± 12 nm; a TK 590 dichromatic mirror and a K 610 barrier filter to select the emitted red fluorescence. The coefficient of variation (CV) for the G0/G1 peaks was evaluated as the width at half maximum of the peak divided by the peak mean channel and the factor 2.35.

For ploidy evaluation, BM cells were analysed either alone or after they had been mixed with an aliquot of PBL, which was employed both to calibrate the instrumentation and as a diploid standard. Aneuploidy was estimated from the DNA-index (DNA-I) value, i.e. the ratio between the model channel of the G0/G1 peak of the sample under study and the modal channel of the G0/G1 peak of the reference standard. For a diploid cell population the DNA-I = 1.00. According to an international convention (Hiddeman *et al*., 1984), only the samples which gave at least two separate G0/G1 peaks were considered aneuploid.

*DNA/p21 flow cytometry*

Bivariate FCM (FITC-green *vs* PI-red) was performed with a FACS Star Cell Sorter (Becton Dickinson FACS Systems, Sunnyvale, CA, USA). Excitation of the FITC-labelled cells and the DNA-associated PI was accomplished with an Innova 90-5 argon ion laser (Coherent, Palo Alto, CA, USA) tuned to 488 nm and operated at 300 mW. Emitted fluorescence was split into two bands (green and red) by means of a dichromatic mirror DM 560: green fluorescence was measured through an interference filter 525 (30 nm h.b.w.), while the red was measured after a long pass filter 620. Instrumentation setting and calibration were performed daily using 1.33 $\mu$m fluorescent microspheres (Polysciences, Warrington, PA, USA).

Data were collected (on a log scale but also converted to a linear scale for comparing differences between experiments) with a Consort 30 (Becton Dickinson FACS System) software program running on a dedicated Hewlett Packard computer and displayed as two-parameter contour density plots. Sequential contours represented increasing isometric equivalents of 5, 15, 30, 60, 120 and 200 cells. A total of 20,000 cells were analysed for each contour plot.

The expression of the antigens studied in the G0/G1, S and G2-M phases of the cell cycle was evaluated from the mean green fluorescence intensity of the corresponding FITC-labelled antibody (inside operationally gated areas) and expressed as mean channel numbers. The same green fluorescence values for the corresponding boxes in matched control samples were also calculated. The 'net' fluorescence intensity associated with the specific staining was calculated by subtracting the nonspecific fluorescence.

*Statistical analysis*

The correlations between Ha-*ras* p21 fluorescence levels and other parameters were examined by the Student's $t$ and $\chi^2$ tests.

**Figure 1** Representative histograms of flow cytometric DNA distribution analysis in peripheral blood lymphocytes, in diploid and aneuploid MM.

### Results

The results obtained are shown in Figures 1–6.

### *DNA flow cytometry*

BMPC% of samples used for FCM analysis ranged from 5 to 18% (median 9%) in MGUS and from 29 to 95% (median 55%) while it was lower than 3% in all normal BM samples. All samples were evaluable by FCM and high-quality DNA profiles were obtained. The values of CV ranged from 2.2 to 3.3 (median = 2.5) for the control samples and from 2.5 to 4.2 (median = 2.8) for the G0/G1 peak of the patients' bone marrow samples (Figure 1).

All normal and MGUS BM samples had diploid DNA content (i.e. the DNA-I of the G0/G1 peak ranged from 0.95 to 1.12, with no additional peak when normal WBC were added to the BM population as an internal standard). Among MM, 21/29 (72.4%) had a single-cell population with diploid DNA content and 8/29 (27.6%) had two cell clones, one diploid and the other hyperdiploid (the median DNA-I of the hyperdiploid clone was 1.50 and the range was from 1.21 to 1.92).

### *DNA/p21 flow cytometry*

Cell clumping was negligible and bright, specific immunofluorescent staining (from FITC-labelled antibodies) was obtained. The fluorescence intensity of tumour cells sequentially treated with sheep anti-p21 *ras* antibody and FITC-labelled goat anti-sheep IgG antibody was strikingly higher than in control samples, i.e. samples of cells exposed to NSS and labelled second antibody or to second antibody only.

Figures 2–5 show representative cytograms of the bivariate analysis of the p21 oncoprotein levels (expressed as mean fluorescence of the G0/G1 peak in arbitrary units) versus the DNA content in normal BM cells, in MGUS and in diploid and aneuploid MM.

In normal and MGUS BM, the p21 oncoprotein level of the G0/G1 cells was consistently low and uniform along the cell cycle (Figures 2 and 3).

In diploid MM, the expression of p21 was more

**Figure 2** Dual staining of p21 Ha-*ras* protein expression and DNA content in normal bone marrow cells.

**Figure 3** Correlated DNA/Ha-*ras* p21 FCM analysis of bone marrow cells from MGUS. The p21 oncoprotein expression is slightly higher than in the normal bone marrow and again evenly distributed along the cell cycle.

heterogeneous. The p21 level was increased (with respect to the level observed in normal and MGUS BM) in at least a part of the G0/G1 cells and these high levels were maintained in the S and G2-M phase cells (Figure 4).

In MM with both diploid and hyperdiploid plasma cells, the p21 expression was distinctly greater in the cell population with higher DNA content than in the diploid population (Figure 5). The median value (in arbitrary units) of p21 expression for the hyperdiploid clone was 40.5 (range 24–57) which was higher than that found in diploid MM (median 20; range 9.5–30; $P < 0.005$). These values were both significantly higher than those found in MGUS (median 7.5; range 3.5–11; $P < 0.005$) and normal BM (median 6.1; range 2.0–8.3; $P < 0.005$) (Figure 6).

**Figure 4** Same analysis performed in diploid MM. The p21 oncoprotein expression is low and uniformly expressed along the cell cycle and, again, there is no clear correlation with a particular phase of the cycle.

**Figure 5** Bone marrow cells from a patient with MM in active phase and with abnormal DNA content. The histogram on the left shows two cell populations with different DNA content and the cytogram on the right their p21 Ha-*ras* expression. The population with higher DNA content also has higher p21 Ha-*ras* expression.

*Correlation between p21 expression and clinical and laboratory parameters in MM*

The p21 expression was greater in patients with advanced (stage III) than in earlier MM (stages I + II) ($P < 0.005$) and it was directly related to BMPC infiltration ($r = 0.7$; $P < 0.005$). No differences in p21 expression were found to be dependent on patient age, type of MC, phase of the disease (presentation or relapse), haemoglobin, levels of serum calcium, type of MC, β2-microglobulin, thymidine kinase or proliferative activity (evaluated as percentage of plasma cells labelled with *in vitro* BUDR and with the monoclonal antibody Ki-67).

**Figure 6** p21 Ha-*ras* expression levels in normal bone marrow, in MGUS in diploid and in aneuploid MM.

### Discussion

Our data suggest that Ha-*ras* is involved in the malignant plasma cell transformation that distinguishes MM from MGUS. In fact, the p21 oncoprotein coded by this oncogene is overexpressed in MM (as a confirmation that this oncogene is activated in MM) (Neri *et al.*, 1989), but not in MGUS, where malignancy is not obvious. Other molecular mechanisms may account for the DNA rearrangement leading to the production of monoclonal immunoglobulin, which is the common feature of both MM and MGUS.

Intact proto-oncogenes normally affect cell growth and/or differentiation by producing regulatory proteins (that often share some specificity for individual cell types and cell cycle states). Alteration of oncogene structure may cause malignancy, due to overproduction, increased specific activity or deregulated synthesis of these proteins (Cooper, 1982; Bishop, 1983; Varmus, 1984). The usual techniques for studying oncogene structure and/or function include blotting studies of DNA or of its RNA message and investigation of the biochemical function of protein products. Immunoblotting studies indicate that *ras* genes are probably involved in (normal) growth control (Shih & Weeks, 1984; McGrath *et al.*, 1984; Sweet *et al.*, 1984; Bar-Sagi, 1989) and that their alteration plays a role in causing several types of human haematological and solid tumours (Viola *et al.*, 1985; Thoret *et al.*, 1984; Lundy *et al.*, 1986; Thein *et al.*, 1986; Liu *et al.*, 1987; Bos *et al.*, 1987; Senn *et al.*, 1988; Pedersen-Bjegard *et al.*, 1988; Hirai *et al.*, 1988).

The technique we used evaluates the presence of an oncogene product in the cells without furnishing data either on the oncogene structure or on the product function. However, there are several advantages with this technique. Based on a MoAb against the oncogene product, bivariate FCM quantitates its expression simultaneously with DNA distribution, and this allows protein expression to be determined both in different cell cycle phases and in distinct cell populations identified according to their DNA content (Danova *et al.*, 1988). There have been technical difficulties in obtaining good specific fluorescences for both the MoAb against the protein and good DNA resolution, but they were overcome with the fixation-permeabilisation technique we utilised (Giordano *et al.*, 1989).

The median p21 expression (that was observed in all cell cycle phases) was low in normal and in MGUS BM and distinctly greater in diploid and especially in hyperdiploid

MM cells (where Ha-*ras* has already been found to be activated frequently) (Neri *et al*., 1989; Tsuchiya *et al*., 1988). A suggestion is that Ha-*ras* alteration is involved in the molecular events leading a plasma cell (already monoclonally determined to MC production in both MGUS and MM) to become a malignant cell as well.

There are several indications that plasma cells progressively change their biological properties from those of normal to MM cells. In MGUS, plasma cells are already abnormal in that they secrete a MC, but they are not malignant. The subsequent malignant transformation of MGUS into MM is a well known event. Finally, clonal selection occurs during the course of MM, with hyperdiploid clones becoming predominant (whereas they had been percentually small at diagnosis) or with the appearance of new hyperdiploid clones (Montecucco *et al*., 1984). Data from our study indicate that p21 expression progressively increases from MGUS to diploid, to hyperdiploid MM, thus paralleling the progressively malignant behaviour of plasma cells.

There are possible clinical implications for p21 expression studies. In our cases, the p21 expression was greater in advanced (stage III) MM and closely related with BMPC infiltration. Alpha-interferon reverses a number of transformed, tumorigenic cell lines (Brouty-Boyè *et al*., 1985) and some reversions are associated with a significant reduction in the expression of the oncogenes implicated in the transformation process (Lin *et al*., 1983; Samid *et al*., 1984). If we speculate that the effectiveness of this agent in MM is due to a similar mechanism, its activity may be evaluated with the FCM method we employed to detect p21 expression. Of course, the clinical meaning of both the presence and the degree of p21 expression in plasma cells dyscrasias, and especially in MM, needs large studies for final confirmations.

At the time of preparation of this manuscript, Dr M. Danova was Visiting Fellow at the National Institute for Cancer Research, I.S.T. (Laboratory of Biophysics and Service of Medical Oncology), Genova, Italy, supported by a grant from I.R.C.C.S. San Matteo, Pavia. Dr M. Giordano was a recipient of a fellowship from the 'Ferrata-Stori' Foundation, Pravia. Research supported by C.N.R. (Consiglio Nazionale delle Ricerche, Roma, Progetto Finalizzato Oncologia, grant no. 88.01422.44), by A.I.R.C. (Associazione Nazionale per la Ricerca sul Cancro, Milano) and by I.R.C.C.S. Policlinico San Matteo (Pavia).

### References

ANDREEFF, M., SLATER, D.E., BRESSLER, J. & FURTH, M.E. (1986). Cellular ras oncogene expression and cell cycle measured by flow cytometry in hematopoietic cell lines. *Blood*, **67**, 676.

BARLOGIE, B., EPSTEIN, J., SELVANAYAGAM, P. & ALEXANIAN, R. (1989). Plasma cell myeloma – new biological insights and advances in therapy. *Blood*, **73**, 865.

BAR-SAGI, D. (1989). Ras proteins: biological effects and biochemical targets. *Anticancer Res*., **9**, 1427.

BISHOP, J.M. (1983). Cellular oncogenes and retroviruses. *Ann. Rev. Biochem*., **52**, 301.

BOS, J., VERLAAN-DE VRIES, M., VAN DER EB, A.J. & 4 others (1987). Mutations in N-ras predominate in acute myeloid leukemia. *Blood*, **69**, 1237.

BROUTY-BOYÈ, D., WYBIER-FRANQUI, J., CALVO, C., FENTEUN, J. & GRESSER, I. (1985). Reversibility of transformed phenotype. Effects of long term interferon treatment on 3H/10T1/2 cells transformed by methylcolanterne and SV40. *Int. J. Cancer*, **34**, 107.

COOPER, G.M. (1982). Cellular transforming genes. *Science*, **218**, 801.

DANOVA, M., RICCARDI, A., GIORDANO, M. & 4 others (1988). Cell cycle-related proteins: a flow cytofluorometric study in human tumors. *Biol. Cell*, **64**, 23.

DURIE, B.G.M. & SALMON, S.E. (1975). A clinical staging system for myeloma: correlation of measured myeloma cell mass with presenting clinical features, response to treatment, and survival. *Cancer*, **36**, 842.

GIORDANO, M., DANOVA, M., RICCARDI, A. & MAZZINI, G. (1989). Simultaneous detection of cellular ras p21 oncogene product and DNA content by two parameter flow cytometry. *Anticancer Res*., **9**, 799.

GOULD, J., ALEXANIAN, R., GOODACRE, A., PATHAK, S., HECHT, B. & BARLOGIE, B. (1988). Plasma cell karyotype multiple myeloma. *Blood*, **71**, 453.

GREIPP, P.R. (1989). Monoclonal gammopathies: new approaches to clinical problems in diagnosis and prognosis. *Blood Rev*., **3**, 222.

HIDDEMAN, W., SCHUMANN, J., ANDREEF, M. & 6 others (1984). Convention on nomenclature for DNA cytometry. *Cytometry*, **5**, 445.

HIRAI, H., OKADA, M., MIZOGOUCHI, H. & 4 others (1988). Relationship between an activated N-ras oncogene and chromosomal abnormality during leukemic progression from myelodysplastic syndrome. *Blood*, **71**, 256.

KRISHAN, A. (1975). Rapid flow cytofluorometric analysis of mammalian cell cycle by propidium iodide staining. *J. Cell Biol*., **66**, 188.

LIN, S.L., GARBEN, E.A., WANG, E. & 5 others (1983). Reduced synthesis of pp60src and expression of the transformation-related phenotype in interferon-treated Rous Sarcoma Virus-transformed rat cells. *Mol. Cell. Biol*., **3**, 1656.

LIU, E., HJELLE, B., MORGAN, R., HECHT, F. & BISHOP, M. (1987). Mutations of the Kirsten-ras proto-oncogene in human preleukemia. *Nature*, **300**, 186.

LUNDY, J., GRIMSON, R., MISHRIKI, Y. & 4 others (1986). Elevated ras oncogene expression correlates with lymph node metastasis in breast cancer patients. *J. Clin. Oncol*., **4**, 1321.

MCGRATH, J.P., CAPON, D.J., GOEDDEL, D.V. & LEVINSON, A.D. (1984). Comparative properties of normal and activated human ras p21 protein. *Nature*, **310**, 644.

MONTECUCCO, C.M., RICCARDI, A., MERLINI, G.P. & 4 others (1984). Plasma cell DNA content in multiple myeloma and related paraproteinemic disorders. Relationship with clinical and cytokinetic features. *Eur. J. Cancer Clin. Oncol*., **20**, 81.

NERI, A., MURPHY, J.P., CRO, L. & 4 others (1989). RAS oncogene mutation in multiple myeloma. *J. Exp. Med*., **170**, 1715.

PEDERSEN-BJERGAARD, J., JANSSEN, J.W.G., LYONS, J.J., PHILIP, P. & BARTRAM, C.R. (1988). Point mutation of the ras protooncogenes and chromosome aberations in acute nonlymphocytic leukemia and preleukemia related to therapy with alkilating agents. *Cancer Res*., **48**, 1812.

RICCARDI, A., DANOVA, M., MONTECUCCO, C.M. & 4 others (1986). Adult acute non-lymphoblastic leukaemia: reliability and prognostic significance of pretreatment bone marrow S-phase size determined by flow cytometry. *Scand. J. Haematol*., **36**, 11.

SAMID, D., CHANG, E.H. & FRIEDMAN, R.M. (1984). Biochemical correlates of phenotypic reversion in interferon-treated mouse cells transformed by a human oncogene. *Biochem. Biophys. Res. Commun*., **119**, 21.

SENN, H.P., TRAN-THANG, C., WODNER-FILIPOWICZ, A. & 6 others (1988). Mutation analysis of the n-ras proto-oncogene in active and remission phase of human acute leukemias. *Int. J. Cancer*, **41**, 59.

SHIH, T.J. & WEEKS, M.O. (1984). Oncogenes and cancer: the p21 ras genes. *Cancer Invest*., **2**, 109.

STEWART, C.C. (1989). Flow cytometric analysis of oncogene expression in human neoplasias. *Arch. Pathol. Lab. Med*., **113**, 634.

SWEET, R.W., YOKOYAMA, S., KAMATA, T., FERAMISCO, J.R., ROSENBERG, M. & GROSS, M. (1984). The product of ras is a GTPase and the T-24 oncogenic mutant is deficient in this activity. *Nature*, **311**, 273.

THEIN, S.L., OSCIER, D.G., FLINT, J. & WAINSCOAT, J.S. (1986). Ha-ras hypervariable alleles in myelodysplasia. *Nature*, **321**, 84.

THOR, A., HORAN, H.P., WUNDERLICH, D., CARUSO, A., MURARO, R. & SCHLOM, J. (1984). Monoclonal antibodies define different ras gene expression in malignant and benign colonic disease. *Nature*, **311**, 562.

TSUCHIYA, H., EPSTEIN, J., SELVANAYGAM, P. & 4 others (1988). Correlated flow cytometric analysis of Ha-ras p21 and nuclear DNA in multiple myeloma. *Blood*, **72**, 796.

VARMUS, H.E. (1984). The molecular genetics of cellular oncogenes. *Ann. Rev. Genet*., **18**, 553.

UCCI, G., RICCARDI, A., LUONI, R. & 4 others (1987). Serum thymidine kinase and $\beta 2$ microglobulin in monoclonal gammopathies. *Tumori*, **73**, 445.

VIOLA, M.V., FROMOWITZ, F., ORAVEZ, S., DEB, S. & SCHLOM, J. (1985). Ras oncogene p21 expression is increased in premalignant lesions and high grade bladder carcinoma. *J. Exp. Med*., **161**, 1213.

WATSON, J.W. (1986). Oncogenes, cancer and analytical cytology. *Cytometry*, **7**, 400.